# Precipitation during two weeks in spring influences reproductive success of first-year females in the long-lived Natterer's bat

**DOI:** 10.1098/rsos.211881

**Published:** 2022-02-16

**Authors:** Bianca Stapelfeldt, Alexander Scheuerlein, Christoph Tress, Ralf Koch, Johannes Tress, Gerald Kerth

**Affiliations:** ^1^ Universität Greifswald Zoologisches Institut und Museum, Greifswald Mecklenburg-Vorpommern, Germany; ^2^ Fledermausforschungsprojekt Wooster Teerofen e.V., Germany; ^3^ Naturpark Nossentiner/Schwinzer Heide, Germany

**Keywords:** timing of reproduction, precipitation, Chiroptera, body size, life history

## Abstract

Bats are characterized by low reproductive rates in contrast with most of other small mammals. This makes their populations vulnerable when inclement environmental conditions such as cold and rainy weather impair the reproductive success of females. The fine-scale effect of weather on bats, however, remains largely unknown. Using a sliding window analysis approach on an 18-year individualized dataset on six Natterer's bat (*Myotis nattereri*) colonies, we investigated the effect of fine-scale weather conditions on age-specific reproductive success. We found that increased precipitation during a short time window in spring strongly reduced the probability of successful reproduction of first-year (FY) females. Our data suggest that this time window is concomitant with implantation or early pregnancy, before substantial investment into embryo development. In addition, larger FY had higher reproductive success, suggesting that reproduction may be condition dependent in young females. Reproductive success of older females was not affected by either weather or individual parameters. Our results show that changes in precipitation pattern may compromise the reproductive success of FY females. Further studies are needed to better understand the impact of weather conditions on reproductive success in long-lived bats under climate change scenarios.

## 1. Introduction

Species with different life-history strategies can be aligned along a slow–fast continuum, based on decisions of resource allocation into survival or reproduction [1,2]. Small mammalian species are usually situated at the fast end of the continuum, as they typically have short lifespans and early ages at first reproduction. Bats, however, are unusual in this context: despite being small, they are characterized by low reproductive rate and long lifespan which are approximately 3.5 times greater than in non-flying placental mammals of similar size [3].

At the same time, as most other small mammals, bats are income breeders and rely on their current foraging success for reproduction. Especially during lactation, when females spend up to 32% of assimilated energy to produce milk [4], young females, being less experienced foragers, can run into energetic problems [5] and may suffer survival costs [6]. In long-lived species such as bats, delaying first reproduction might enable females to improve their chance of survival and reproductive rate [7]. In a British population of *Myotis daubentonii*, 80% of 1-year-old females bred, which resulted in a 33% lower survival probability of breeders as compared with non-breeding young females. By contrast, in sympatric populations of *M. nattereri*, it was only 57% of the 1-year-old females that reproduced, with no survival costs observed [6].

Costs of reproduction also depend on environmental conditions [8–11]*.* In bats, cold, rainy and windy weather increases energy costs of maintaining body temperature and simultaneously reduces arthropod prey species abundance and activity [12–16]. Consequently, young and inexperienced females may skip reproduction when spring weather conditions are unsuitable [17]. Of course, the effect of weather on reproduction differs among species [18] and regional climatic conditions. In arid areas, exceptionally dry years drive bats into water stress with limited reproductive success [19,20], whereas in regions with generally higher annual rainfall, low precipitation may have positive effects [9]. The season when bats are sensitive to weather is also highly variable [9,17,21]. Jan *et al.* [21] showed that in lesser horseshoe bats (*Rhinolophus hipposideros*), the effect of precipitation on fecundity was negative during April, but positive during October. The same study showed that finer time scales are generally more informative in explaining the impact of weather on bat population dynamics [21]. In addition, elucidating the exact period of weather sensitivity would allow for a better understanding of the mechanisms involved in the reproductive success of bats. Still, most previous studies on the effect of weather on population dynamics analysed larger time frames of months or entire seasons [9,14,17,21–23] and therefore were unable to determine the exact period of weather sensitivity.

In the temperate zone, climate varies dramatically between summer and winter. In early spring, when females emerge from hibernation, they ovulate and fertilize with stored sperm [24]. Under adverse weather conditions, females can roost in larger groups to reduce energy expenditure while staying homoeothermic. Alternatively they can save energy by entering torpor—a physiological state characterized by reduced metabolism [25]. However, if pregnant females spend time in torpor long, they have to face the trade-off that embryo development is reduced and parturition is delayed [26]. This is illustrated by the observations that spring temperatures are a good predictor for parturition date in many bat species of the temperate zone [9,10,27]. But parturition date may also affect juvenile survival. Early parturition is beneficial as it gives juveniles more time to improve their foraging abilities before stocking up on body reserves for their first hibernation [6,11,14,17,18] and promotes the early attainment of sexual maturity in both sexes [8,28]. By contrast, late parturition can lead to lower juvenile survival and lower reproductive success in first-year (FY) females that return from their first hibernation [17]. Additionally, late parturition and cold temperatures during pregnancy and juvenile growth are associated with smaller body size in bats [27,29,30]. This is important for population persistence, as there is evidence that body size is a fitness-relevant trait in bats, with large size being beneficial in some species [31–33], but detrimental in others [27,34].

In our study species, the Natterer's bat (*Myotis nattereri*), a number of up to 80 adult females form maternity colonies from April to October to communally rear their young [35]. Natterer's bats mate in autumn and winter [36] and emerge from hibernation between January and April [37]. Ovulation in temperate-zone bats occurs after emergence from hibernation [24,38]. In Natterer's bats, females give birth to one offspring per year between June and July [39], suggesting that ovulation and embryo implantation occurs around April. Despite their small body size (7–10 g, Dietz *et al.* [39]) Natterer's bats can reach an age of up to 23 years [40].

Here, we study the effect of fine-scale weather conditions on age-specific reproductive success (2003 to 2020) in Natterer's bat colonies using a sliding window analysis approach. Being able to define a precise time window that can be linked to a specific stage in the bats' annual cycle would allow inference on the mechanism involved, and would also inform on the evolvability of the population under climate change. This allows for a more robust prediction of population persistence in the future. We combine this approach with further potential predictors of reproductive success, such as colony size (as a proxy for social thermoregulation), colony membership (as different colonies may have habitats with different quality), females’ body size (as a potential fitness-relevant trait), reproduction in the previous year (to assess costs of previous successful reproduction) and parity (as a measure of individual experience). We surmise that adverse weather conditions affect 1-year-old females more than older individuals, and that this effect is modulated by condition-dependent factors, as mentioned above.

## 2. Methods

### 2.1. Study site and data collection

The study area is situated in the nature park Nossentiner/Schwinzer-Heide in Mecklenburg-Pomerania, northern Germany. Every year since 1990 bat and bird boxes occupied by Natterer's bats were brought to the field station between the end of July and the beginning of September after the juveniles fledged. Individuals were handled and released back into the box. After this procedure, the boxes with the bats were brought back to the original place. During handling, forearm length and body weight were taken, age (juvenile versus adult) and reproductive condition of the adult females (reproducing versus non-reproducing) were assessed and all unmarked bats received a forearm band. Juveniles were identified by unfused metacarpal-phalangeal epiphyseal gaps [30]. To ensure that only the size of fully matured individuals is analysed, we used only forearm measurements of the first re-capture as an adult after an individual's first hibernation. Reproductive condition was assessed visually after the lactation period, end of July and early August: females with bare skin around extended nipples were classified as ‘reproducing’ and females with inconspicuous fur-covered nipples were classified as ‘non-reproducing’. Please note, that mothers that lost their pup very early during pregnancy or lactation were classified as ‘non-reproducing’. Thus, ‘reproducing’ means that females were able to raise an offspring at least to the fledgling stage. Handling and marking were carried out by applying standard bat handling procedures (e.g. [8,9,17,27,34,41]) and under licence from the appropriate nature conservation authorities.

We restricted our dataset to the years 2003 until 2020 because sample sizes of individuals and recapture rates were low during establishment of the study population, and the weather data had large gaps before 2003.

### 2.2. Statistical analysis

#### 2.2.1. Reproductive rates

We calculated reproductive rates separately for every year and each age class. FY females had been marked as juveniles the previous year and were recaptured in the year after their birth year. Second-year-plus (SY+) females comprised all females that survived at least two winters. For females that had not been marked as juveniles, we excluded the observations of reproductive success of the first year they had been captured, as their age class (FY or SY+) could not be reliably assessed from external features. To calculate age-class-specific reproductive rates, we divided the number of reproducing females by the number of all females (reproducing + non-reproducing) for each year.

For all following analyses, we fitted models with binomial distribution on reproductive condition as a response variable, (reproducing = 1) or not (non-reproducing = 0).

#### 2.2.2. Sliding window analysis

To detect the most sensitive time window when weather parameters affect reproductive success, we applied a sliding window analysis with the package climwin [42]. In this approach, models with all possible time windows are fitted and compared among each other using the information-theoretic model selection criteria AICc and proportion of models, belonging to the 95% confidence set for each tested weather parameter [42,43]. We obtained climate data from the archive of the DWD meteorological station Goldberg (ID 01694).

We analysed the effect of *mean temperature*, *minimum temperature* and *cumulative precipitation* per 24 h period (DST 05.00–04.59), and *cumulative precipitation* and *mean wind speed* during night-time (DST 22.00–04.59) (electronic supplementary material, appendix and table S1). As Natterer's bats return to the study area during April, we designed the sliding window approach starting on 1 April and ending at 31 July, after juveniles were weaned (FY: *N* = 329, SY+: *N* = 1463).

We considered auto- and collinearity of the weather parameters and confirmed the best-supported model with 150 randomizations (see electronic supplementary material, figure S1).

#### 2.2.3. Effects on reproductive success

We analysed the effect of relevant weather parameters (identified in the sliding window analysis), together with additional predictors, on the reproductive success of FY using generalized linear models (GLM; family = binomial). Additional predictors used were year of observation, forearm length (*hereafter* body size), colony ID and colony size (for details see electronic supplementary material, section *colony size and recapture rate*). Individuals without body size information were excluded. This reduced the FY dataset to 292 females. Model selection was performed using the AIC.

In a next step, we investigated potential drivers for SY+ females' reproductive success by fitting generalized linear mixed models (GLMMs) with ring ID as random effect (package lme4, [44]). Year, colony ID, colony size, body size, minimum parity (the number of reproductive events a bat experienced) and whether a bat reproduced the year before (previous reproduction) were entered as fixed predictors. To avoid empty cells in the data matrix, the variables *minimum* parity, colony size and body size were recoded as quantiles and treated as factors. We fitted *minimum* parity, because some individuals were not caught in each year of their lifetime and reproductive status could not be assessed. If an individual was not recaptured in 1 year, we also needed to exclude the observation in the following year for that individual, as in this case, it was not possible to determine the value for the variable ‘previous reproduction’. Both observations were then treated as ‘censored’ and omitted from analysis. If the individual was then recaptured in at least 2 consecutive years thereafter it entered the analysis from there onwards again. This resulted in a dataset with *N* = 1029 annual observations (bat-years) obtained from 402 SY+ individuals.

To ensure that the analysis was not biased by censored observations, we repeated the SY+ analyses (climwin and GLMMs), exclusively using females with minimum 90% complete capture histories (for details, see electronic supplementary material, section *SY+ analysis with females having high recapture rates*). This restricted analysis produced qualitatively similar results as the full analysis. We therefore conclude that the analyses on SY+ reproductive success are robust.

To measure the effect size and the degree of certainty of our model estimates, we calculated the 95% credibility intervals (‘effectsize’ function from the package effectsize [45]).

## 3. Results

### 3.1. Reproductive success across years

**Figure 1.**
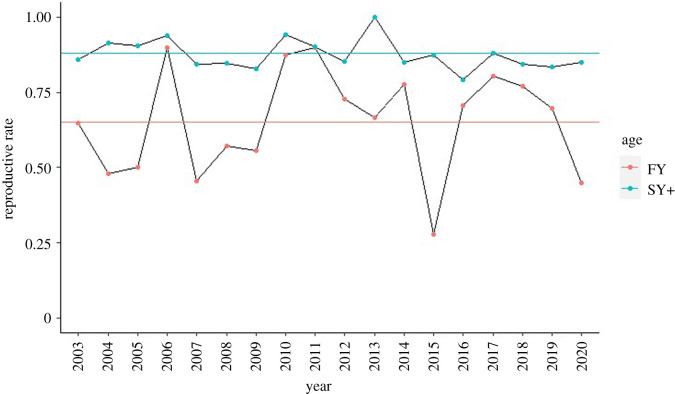
Annual reproductive rate of FY females with a mean of 65% (±18%) (horizontal red line) and of SY+ with mean of 88% (±5%, horizontal blue line).

FY females had a mean annual reproductive success of 65% (±18% s.d.), which was lower and varied much stronger across the years compared with SY+ females with a mean annual reproductive success of 88% (±5% s.d., figure 1) (for reproductive success since 1990 see electronic supplementary material, figure S2).

### 3.2. Environmental and individual effects on reproductive success

**Figure 2.**
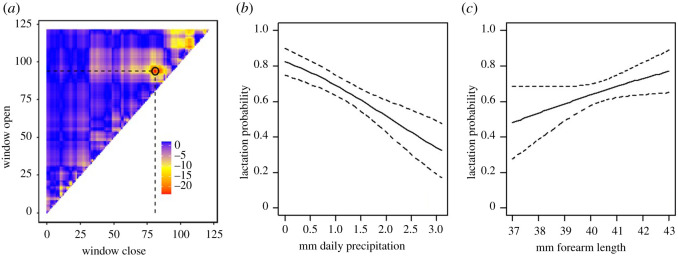
(*a*) Heat map of ΔAICc values for all fitted climate windows which display a clear peak at the precipitation sensitive time (28 April–11 May) for the FY females; (*b*) reproductive success in FY females decreases with increasing spring precipitation (*c*) and increases with forearm length (lines present predicted response from the best model).

In FY females, we found that of all tested weather parameters only daily cumulative precipitation (hereafter *spring precipitation*) during a narrow time window (28 April–11 May) had a negative effect on reproductive success (electronic supplementary material, table S1; figure 2*a*). This was confirmed in the subsequent GLM analysis where spring precipitation, together with body size, were the only factors in the best model explaining the effect on reproductive success in FY females, (see electronic supplementary material, table S3). Spring precipitation had a negative effect on reproductive success (table 1 and figure 2*b*), while larger FY females tended to have a higher reproductive success (table 1 and figure 2*c*).

**Table 1.** Best-fit GLM estimating the effect of spring precipitation (28 April–11 May) and body size (forearm length) on the reproductive success of FY with corresponding credibility intervals (95% CI) estimating model uncertainty.

fixed effect	estimate	s.e.	*z*-value	*p*-level	95% CI
intercept	−7.0651	4.7867	−1.479	0.1399	0.43, 0.93
spring precipitation	−0.7329	0.1807	−4.055	<0.001	−0.80, −0.28
body size	0.2130	0.1189	1.792	0.0731	−0.02, 0.49

By contrast, reproductive success in SY+ females was not affected by the parameters we tested: the sliding window analysis showed that none of the tested weather parameters had an effect (electronic supplementary material, table S1), and the GLMM showed that the only parameter included in the best model, previous reproduction, was not significant (estimate = 0.2372, *p* = 0.373, 95% CI −11, 0.29; see electronic supplementary material, table S4).

## 4. Discussion

### 4.1. Different reproductive success in FY and SY+ females

FY females had lower, and more variable reproductive success across the years than older (SY+) females, which confirms previous findings in *M. nattereri* and other *Myotis* species [6,17]. Bats show remarkable parental investment for mammals of their small size [46] with a long gestation period [47], neonates that make up to 28% of maternal body weight [48] and an extended period of parental care [46]. As income breeders, female bats have to fine tune their increased energy demand during reproduction to variable environmental conditions that affect both food supply and thermal conditions [49]. Individuals of long-lived species like *M. nattereri* have multiple opportunities for reproduction during their lifetime, why reproductive restraint may be an option in a year with adverse conditions. It is well known that young bats are inexperienced and inefficient foragers [5,17]. It seems therefore plausible that FY females are more likely to forgo reproduction than older females, especially under adverse environmental conditions [6,7].

### 4.2. Impact of rain and body size on reproductive success

Elevated levels of spring precipitation reduced reproductive success in FY females in our study population. In some other studies on bats, high precipitation was also linked to low levels of reproduction with delayed gestations, smaller newborns and reduced juvenile growth rates [5,9,13]. Further studies found detrimental effects on reproduction in response to lower temperatures [10,27,30]. For both parameters—low temperatures and rainfall—it is suggested that negative effects are caused by an energetic bottleneck due to higher thermoregulatory costs and increased foraging costs due to a reduced activity of arthropod prey species [12–14]. As a gleaning species, Natterer's bats are not dependent on flying insect activity and have been reported to even forage during the hibernation period [50]. Increased precipitation reduces the effective range of bat's echolocation [51] in addition to increased thermoregulatory costs [15]. As a result, during rainy weather periods body condition of some FY females may reach a critical low and they may skip reproduction*.* From other bat species, it is known that in an arid climate, the effect of precipitation on reproductive success can even be positive, because pregnant and lactating females have much higher water demand than non-reproductive females [52]. Thus, in regions where rain is necessary for water availability, low precipitation levels drive bats into water stress and can therefore cause low reproductive rates [19,20]. However, our study area is localized in a temperate region with many lakes close to all maternity roosts (within 1.5 km) that ensure water availability, even in dry years.

Most surprisingly, and not shown before in other studies that used less fine-scaled indicators of environmental conditions [17], the sensitive time window affecting FY females' reproduction comprised only a two-week period at the end of April and the first 11 days of May. Although specific studies on the timing of ovulation in *M. nattereri* are lacking, it is around this time that European vespertilionid bats ovulate [38] and fertilize the eggs with sperm obtained in the mating season in the previous autumn [36]. Because phenological events such as the timing of ovulation and implantation as well as length of gestation can vary with environmental conditions and the associated use of torpor [9,53–56], the exact reproductive stage of young females during the sensitive window is hard to know. During poor weather conditions, bats can abort blastocysts or embryos [55,56], and this probably occurs at an early stage of development [13]. Thus, we assume that the sensitive window we identified in our analysis comprises implantation and early embryo stages. If FY females gave birth late because they had to spend much time in torpor during rainy weather, the remaining time period for juveniles to grow and accumulate fat resources required for successful hibernation would have shortened. Late-born male juveniles would have had a reduced chance of reaching sexual maturity in their first autumn, and female juveniles would have had reduced reproductive rates the following year [8,14,17,28]. Both sexes alike would have had lower chances of surviving hibernation if they were late-born. It seems plausible that it has long-term fitness benefits for FY females to skip reproduction when births otherwise would happen to be late in the year.

At the same time, we found a positive effect of body size on the reproductive success of FY females. Previous studies have shown that both benign environmental conditions and an early birth date lead to larger body sizes in bats [27]. Larger FY females may thus be in better body conditions after hibernation than smaller FY females and are therefore more likely to initiate pregnancy. This finding suggests that both intrinsic and extrinsic factors influence reproductive success in FY female Natterer's bats.

Interestingly, older females have high reproductive success regardless of weather conditions or individual parameters, such as body size or if they had a birth event in the previous year. It seems plausible that their experience might allow them to better cope with adverse conditions regardless of their body size.

In comparison with colonies of similar forest-living species like Bechstein's bats (*Myotis bechsteinii*), where the colony size is likely to influence social thermoregulation [25], colonies of Natterer's bats are more numerous [35]. In our study population, colonies may be always numerous enough to buffer adverse temperature effects (mean 33 adult females; maximum 61 adult females in our study population), which may explain why we did not find an effect of temperature on reproductive success either in FY or in older females.

According to recent climate change scenarios, precipitation during winter and spring will most likely increase in northeastern Germany [57]. In addition, the number of days with extreme precipitation events is supposed to rise, with the strongest increase of heavy rain events during winter and spring [57]. This may lower foraging opportunities for Natterer's bats during times when prey is scarce anyway and therefore may reduce winter survival and reproductive success.

## 5. Conclusion

Natterer's bats' life-history decisions are characteristic for long-living organisms with delayed first reproduction under unsuitable conditions [7]. For FY females in our study population, such unsuitable conditions are met by higher precipitation. Rain is known to reduce foraging efficiency and simultaneously dramatically increases energy expenditure if bats become wet [15]. The time when FY females skip reproduction is probably around the timing of implantation, before any considerable resource allocation into embryo development occurred. Moreover, for FY females, body size is a fitness-relevant trait and large size is beneficial in terms of reproduction. This raises the demand for further studies on the impact of individual characteristics on individual fitness in bats, and of the impact of changing weather condition under climate change scenarios.

## Data accessibility

Data and Script are provided as electronic supplementary material, tables. The data are provided in the electronic supplementary material [58].
